# Cervical Cancer Screening Among Rural Women in Ernakulam District, Kerala: A Cross-Sectional Study

**DOI:** 10.7759/cureus.100961

**Published:** 2026-01-06

**Authors:** Melvi Johnson, Teena Mary Joy, Amina Rasheed, Brilly M Rose, Aswathy S

**Affiliations:** 1 Community Medicine, Amrita Institute of Medical Sciences and Research Centre, Kochi, IND

**Keywords:** awareness, cervical cancer, pap smear, screening, symptoms of cervical cancer

## Abstract

Background

Cervical cancer (CC) remains a significant public health concern globally, especially in rural areas where screening rates are notably low. This study aims to assess CC screening practices, awareness, and associated factors among rural women in Ernakulam district, Kerala.

Methodology

A community-based, cross-sectional study was conducted from January 2024 to March 2024. A sample of 513 women aged 25-60 years was selected using multistage sampling. Data were collected using a semi-structured questionnaire and analyzed using SPSS version 20 software (IBM Corp., Armonk, NY, USA).

Results

The prevalence of CC screening was found to be 9.2%, with 86.2% of women expressing willingness to undergo future screening if provided free of charge. Nearly half (48.7%) of the study population had average or above average awareness of CC and its screening practices. Factors associated with increased screening uptake included age of more than 41 years, those from the above poverty line category, and those living in nuclear families.

Conclusions

This study provides valuable insights into the practices of CC screening and awareness among rural women in Ernakulam district, Kerala. Targeted educational interventions and improved access to screening services are recommended to address gaps.

## Introduction

Cervical cancer (CC) ranks as the fourth most prevalent cancer among women worldwide, with approximately 660,000 new cases reported in 2022 [1]. In India, it ranks as the second most common cancer among women, accounting for 9.4% of all cancer cases [2]. India has seen a significant drop in both the number of cases and deaths from CC over the years. The age-standardized incidence rates vary widely from state to state, with Tamil Nadu having the highest rate at 19.5 and Jammu and Kashmir having the lowest at 6.13 [3].

To accelerate the elimination of CC as a public health problem, the World Health Organization (WHO) has launched a global strategy that aims to reduce the incidence below 4 cases per 100,000 women-years in every country by 2030 [4]. WHO established the 90-70-90 targets, which include achieving high coverage rates for humanpapilloma virus (HPV) vaccination (90% of girls fully immunized with the HPV vaccine by the age of 15 years), screening (70% of women screened using a high-performance test by 35 years of age and again by 45 years of age), and treatment of precancerous lesions, as well as management of cancer (90% of women with pre-cancer treated and 90% of women with invasive cancer managed) by 2030 [4].

Research shows that even one round of CC screening can significantly lower both incidence and mortality [5]; however, screening rates vary widely between countries. Despite WHO recommendations, globally two-thirds (64%) of women aged 30-49 years have never been screened for CC [6]. The screening coverage is particularly low in the low- and middle-income countries, which can be attributed to a lack of healthcare facilities and national screening programs [7,8]. In India, the proportion of women undergoing CC screening is alarmingly low, varying from 1.97% [9] nationally to as low as 0.2% in states such as West Bengal and Assam and peaking at 10.1% in Tamil Nadu [10]. Various studies have shown that factors such as age, education, employment status, number of sexual partners, knowledge about CC, and attitude significantly influence screening practices [11,12].

In Kerala, the incidence of CC has been decreasing over the years [13]. Recent studies in Kerala have noted the age-standardized incidence to be 4.3 per 100,000 women from the cancer registry data in Thiruvananthapuram [14]. This decreasing trend provides Kerala with an opportunity to aim for the elimination of CC from the state, which has a Human Development Index almost comparable to that of developed countries [15].

Given that a significant portion of India’s population resides in rural areas, understanding CC screening practices among rural women is vital for creating targeted interventions to improve access and uptake. Furthermore, few studies focus on CC screening and awareness in rural areas of Kerala. Therefore, the present study aims to assess the prevalence of CC screening practices among women residing in rural areas of Ernakulam district, Kerala. In addition, it aims to assess the level of awareness regarding CC and its screening, as well as to determine the factors associated with the uptake of CC screening.

## Materials and methods

A community-based, cross-sectional study was conducted among women aged 25-60 years in the rural areas of Ernakulam district from January 2024 to March 2024. The study was approved by the institutional ethical committee (ECASM-AIMS-2024-187; dated May 28, 2024). Informed consent was obtained from each participant before data collection. Women who had been diagnosed with CC or had undergone a hysterectomy were excluded from the study. The sample size was calculated based on a study from central Kerala [16], in which the prevalence of anytime CC screening among women in rural communities was 8.3%. Assuming a 95% confidence level and an absolute precision of 4%, the minimum sample size was calculated to be 183. After applying a design effect of 2 for multistage sampling and adjusting for a non-response rate of 20%, the final required sample size was approximately 458. However, data were collected from 513 women to improve the precision of estimates.

**Figure 1:**
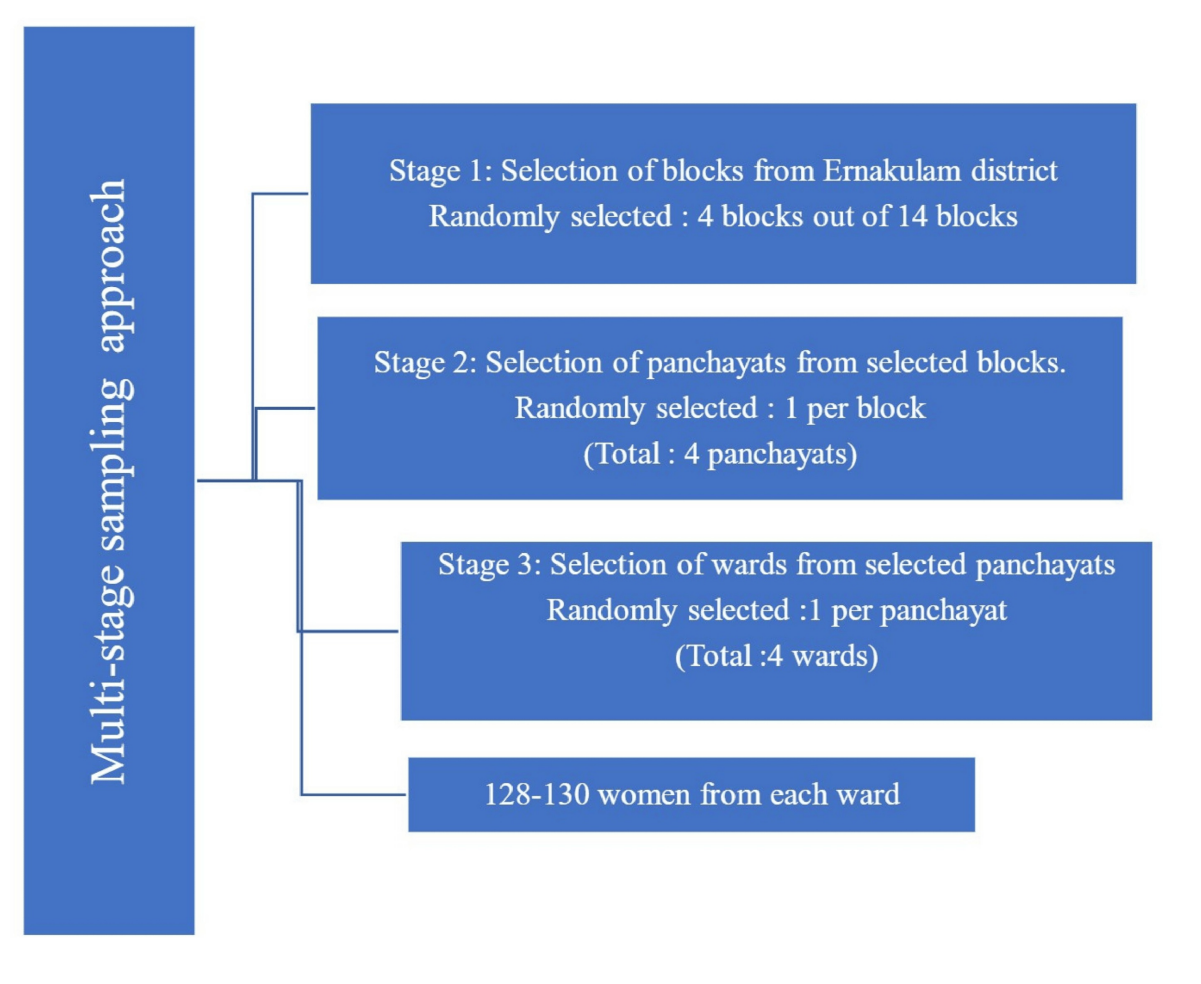
Flowchart illustrating the multi-stage sampling technique.

The study utilized a multistage random sampling approach. First, four blocks were chosen at random from the 14 blocks in Ernakulam district. Following this, one panchayat was randomly selected from each of the chosen blocks. Then, one ward was randomly picked from each selected panchayat. Systematic random sampling was employed to select the study participants from each of these four wards. The total female population in each ward was obtained from the updated electoral rolls and ranged from 474 to 693. The sampling interval was determined as 4. The second woman from the list was randomly selected as the first participant, and after adding the sampling interval of 4, the other participants were selected. Using this method, we selected 128-130 women from each ward (Figure 1).

A semi-structured questionnaire adapted from a prior study from South India [17] served as the study tool for the current study. The questionnaire had two sections. The first section gathered sociodemographic data such as age, gender, education, occupation, marital status, age at marriage, parity, age at first childbirth, and socioeconomic status (SES). SES was determined by the color of the ration card. In Kerala, households are categorized as above the poverty line (APL) and below the poverty line (BPL) by their household income. White and blue cards belong to the APL category, and yellow and pink cards belong to the BPL category. The second part focused on assessing their knowledge regarding CC and its screening methods. Knowledge about CC, screening, and prevention was evaluated using a 16-point scale [18]. Questions had multiple responses, and each correct response was assigned a score of 1, while incorrect answers received 0 points. The total score ranged from 0 to 16. Scores were given for various aspects, including awareness of CC, recognition of CC symptoms, risk factors, knowledge of preventive measures, familiarity with treatment options, awareness of screening methods, eligibility criteria for screening, recommended frequency of screening, knowledge of screening locations, and awareness of vaccination against CC. Awareness was assessed using a structured scoring system, in which a score of <8 was considered below-average awareness for this study.

The data were then entered into Microsoft Excel (Microsoft Corp., Redmond, WA, USA) and analyzed using SPSS version 20.0 (IBM Corp., Armonk, NY, USA). Descriptive statistics were computed as proportions for categorical variables and means (with standard deviations) for continuous variables. Univariate analysis was performed using the chi-square test to identify factors associated with better uptake of CC screening. Variables that showed a statistically significant association in univariate analysis were subsequently included in a multivariable logistic regression model to identify independent predictors of CC screening uptake. A p-value <0.05 was considered statistically significant.

## Results

The mean age of the study population was 41 ± 10 years, with ages ranging from 25 to 60 years. About 53% of the participants had a graduate-level education or above. More than half (56.3%) of the women were employed, and about 55% of the study population belonged to the APL category based on the color of the ration card. The majority of women were married (90.3%), and the mean age of marriage among the study population was 23 ± 2.8 years (Table 1).

**Table 1:** Sociodemographic details of the study population.

Variables	Categories	Frequency	Percentage
Age	≤41 years	266	51.9
	>41 years	247	48.1
Education	Up to higher secondary	241	47
	Graduate and above	272	53
Occupation	Unemployed	282	55
	Employed	231	45
Religion	Christian	149	29
	Hindu	274	53.4
	Muslim	89	17.3
	Others	1	0.2
Marital status	Unmarried	50	9.7
	Married	437	85.18
	Widow	24	15.68
	Divorced	2	0.38
Age at marriage (n = 463)	≤21 years	128	27.6
	>21 years	336	72.6
	Unmarried	50	10.8
Age at first childbirth (n = 463)	≤23 years	135	29.2
	>23 years	304	65.7
	Never got pregnant	24	5.2
Number of children (n = 463)	No children	24	5.2
	1–2 children	371	80.1
	More than 2 children	68	14.7
Type of family	Nuclear family	286	55.8
	Three-generation family	209	40.7
	Joint family	18	3.5
Family history of cervical cancer	Yes	29	5.7
	No	484	94.3
Received information about cervical cancer from healthcare workers	Yes	175	34.1
	No	338	65.9
Family support in visiting a doctor for gynecological symptoms	Yes	457	89.1
	No	56	10.9
Feels shy to share about gynecological symptoms	Yes	39	6
	No	474	92.4
Presence of symptoms like abnormal vaginal bleeding/foul smelling discharge	Yes	42	8.2
	No	471	91.8

Among the study population, the prevalence of ever undergoing CC screening was found to be 9.2% (95% confidence interval (CI) = 8.45, 9.95). On univariate analysis, a significant association was found between age, educational qualification, SES by color of ration card, type of family, marital status, recipients of health information from healthcare workers about CC, awareness score, and practice of CC screening (Table 2).

**Table 2:** Factors associated with cervical cancer screening among women. OR = odds ratio; CI = confidence interval

Category	Screened for cervical cancer		Crude OR (95% CI)	P-value
	Yes n (%)	No		
Age				
≤41 years	10 (3.8)	256 (96.2)	0.22 (0.11–0.45)	0.001
>41 years	37 (15.0)	210 (85.0)	—	—
Education				
Up to higher secondary	11 (4.6)	230 (95.4)	0.31 (0.15–0.63)	0.001
Graduate and above	36 (13.2)	236 (86.8)	—	—
Occupation				
Unemployed	18 (8.0)	206 (92.0)	0.78 (0.43–1.45)	0.436
Employed	29 (10.0)	206 (90.0)	—	—
Socioeconomic status (by color of ration card)				
Above the poverty line	42 (14.9)	240 (85.1)	7.9 (3.07–20.35)	0.001
Below the poverty line	5 (2.2)	226 (97.8)	—	—
Religion				
Christian	17 (11.4)	132 (88.6)	—	—
Hindu	22 (8.0)	252 (92.0)	—	—
Muslim	8 (9.0)	81 (91.0)	—	0.698
Others	0 (0)	1 (100)	—	—
Marital status				
Unmarried	0 (0)	50 (100)	1.13 (1.07–1.14)	0.018
Married/Widow/Separated	47 (10.2)	416 (89.8)	—	—
Type of family				
Nuclear	33 (11.5)	253 (88.5)	1.98 (1.03–3.80)	0.036
Others (three-generation/joint)	14 (6.2)	213 (93.8)	—	—
Family history of cervical cancer				
Yes	3 (10.3)	26 (89.7)	1.15 (0.34–3.96)	0.82
No	44 (9.1)	440 (90.9)	—	—
Received health information from healthcare workers about cervical cancer				
Yes	30 (17.1)	145 (82.9)	3.9 (2.08–7.31)	0.001
No	17 (5.0)	321 (95.0)	—	—
Family support in visiting a doctor for gynecological symptoms				
Yes	46 (10.1)	411 (89.9)	6.1 (0.83–45.5)	0.043
No	1 (1.8)	55 (98.2)	—	—
Awareness about cervical cancer and screening				
Less than average(score <8)	11 (4.2)	252 (95.8)	0.25(0.13-0.52)	0.001
Average and above (score *≥*8)	36 (14.4)	214 (85.6)	—	—

In multivariable regression, those aged less than or equal to 41 years (adjusted odds ratio (AOR) = 0.13; p = 0.001; 95% CI = 0.55-0.278) were less likely to undergo screening compared to those above 41 years. Moreover, those belonging to the APL category (AOR = 7.9; p = 0.001; 95% CI = 2.45-23.69), those from nuclear families (AOR = 2.25; p = 0.02; 95% CI = 1.37-6.07), and those who received health information from healthcare workers about CC (AOR = 4.11; p = 0.012; 95% CI = 2.017-8.38) were more likely to undergo screening for CC (Table 3).

**Table 3:** Independent predictors for uptake of cervical cancer screening (n = 513). OR = odds ratio; CI = confidence interval

Variables	Screened		Adjusted OR	95% CI (lower–upper)	P-value
	Yes, n (%)	No, n (%)			
Age					
≤41 years	10 (3.8)	256 (96.2)	0.124	0.055 – 0.278	0.001
>41 years	37 (15.0)	210 (85.0)	1 (Ref.)	—	—
Education					
Up to higher secondary	11 (4.6)	230 (95.4)	0.46	0.186 – 1.145	0.09
Graduate and above	36 (13.2)	236 (86.8)	1 (Ref.)	—	—
Socioeconomic status (by color of ration card)					
Above the poverty line	42 (14.9)	240 (85.1)	7.627	2.455–23.699	0.001
Below the poverty line	5 (2.2)	226 (97.8)	1 (Ref.)	—	—
Received health education from healthcare workers about cervical cancer					
Yes	30 (17.1)	145 (82.9)	4.112	2.017–8.38	0.012
No	17 (5.0)	321 (95.0)	1 (Ref.)	—	—
Type of family					
Nuclear	33 (11.5)	253 (88.5)	2.894	1.37–6.07	0.02
Others (three-generation/joint)	14 (6.2)	213 (93.8)	1 (Ref.)	—	—
Family support in visiting a doctor for gynecological symptoms					
Yes	46 (9.5)	411 (89.9)	4.525	0.544–37.662	0.103
No	1 (1.8)	55 (98.2)	1 (Ref.)	—	—
Awareness about cervical cancer and screening					
Low (<8)	11 (4.2)	252 (95.8)	2.18	0.98–4.86	0.06
Good (*≥*8)	36 (14.4)	214 (85.6)	1 (Ref.)	—	—

Awareness about cervical cancer and its screening

About 83.4% (428 out of 513) of women had heard of CC, and the majority (81%, 348 out of 428) reported media as the source of their information. One-fourth (25.7%, 110 out of 428) of the women were unaware of any symptoms of CC. Among those who were aware, the most frequently reported symptoms were intermenstrual bleeding (82%, 203 out of 247) and foul-smelling discharge (75.3%, 186 out of 247). Most women identified having multiple sexual partners as a significant risk factor for developing CC (71.6%, 177 out of 272). Regarding preventive measures, 62.3% (267 out of 428) of the women identified at least one strategy, with the majority (70%, 187 out of 267) reporting having a single trustworthy partner and a quarter (24.7%, 68 out of 267) considering HPV vaccination as the preventive strategy (Table 4).

**Table 4:** Awareness about cervical cancer among the study participants. *: Multiple responses possible. HPV = human papillomavirus; OCP = oral contraceptive pill

Awareness characteristics	Frequency (%)
Ever heard of cervical cancer	428 (83.4)
Awareness of any symptoms (n = 428)	247 (57.7)
Awareness of any risk factors (n = 428)	272 (63.5)
Awareness of any preventive strategies (n = 428)	267 (62.3)
Symptoms of cervical cancer (n = 247)*	
Intermenstrual bleeding	203 (82.0)
Foul-smelling vaginal discharge	186 (75.3)
Bleeding after intercourse	111 (44.9)
Post-menopausal bleeding	175 (70.8)
Urinary urgency	27 (11.0)
Low back pain and lower abdominal pain	140 (56.6)
Risk factors for cervical cancer (n = 272)*	
Having multiple sexual partners	177 (71.6)
Early age of sexual activity	91 (33.4)
Acquiring the HPV infection	78 (26.3)
Prolonged use of oral contraceptive pills	48 (17.6)
Parity (>5)	26 (9.5)
History of other sexually transmitted diseases	84 (30.8)
Poor menstrual hygiene	86 (31.6)
Preventive strategies for cervical cancer (n = 267)*	
Single trustworthy partner	187 (70.0)
Avoid early age of sexual intercourse	115 (43.0)
Vaccination against HPV	68 (24.7)
Avoid pregnancy at a young age	51 (19.0)
Avoid chronic use of OCP	79 (29.5)
Ever heard of cervical cancer screening tests (n = 428)	149 (34.8)
Ever heard of the HPV vaccine (n = 513)	68 (13.2)
Overall awareness about cervical cancer and screening	
Less than average (score <8)	263 (51.3)
Average and above (score ≥8)	250 (48.7)

About 34.8% (149 out of 428) of women who had heard about CC were aware of the early detection of CC through screening tests. However, about half of these women, 51.6% (77 out of 149), could not name any screening tests, and 46.3% (69 out of 149) of them mentioned the Papanicolaou test (Pap smear) as the screening test. About 14.8% (22 out of 148) reported the age-related eligibility criteria to be 25 years for CC screening, and 39.8% (59 out of 148) mentioned having multiple sexual partners as an eligibility criterion. When inquired about the frequency of screening for CC, more than half (59%, 88 out of 148) of the women were unaware of the timings. After providing all participants with brief information about the disease and available screening options, 86% (441 out of 513) expressed willingness to undergo screening if it were provided free of charge. In addition, 65% (333/513) reported that they would be willing to undergo self-sampling for CC screening if such an option were made available. Among the study participants, 51.3% had below average awareness, and 48.7% had average or above average awareness about CC and its screening.

## Discussion

The prevalence of CC screening in Ernakulam district was found to be 9.2%. The factors for increased uptake of CC screening were found to be age above 41 years, belonging to the APL category, being from nuclear families, and receiving health information about CC from healthcare workers.

The observed prevalence is higher compared to earlier studies conducted in rural Kerala and other parts of India [19,20]. Previous research from Kerala reported screening prevalence ranging from 6.9% [21] in 2012 to 8.3% in 2018. The increasing trend in screening uptake can likely be attributed to proactive community-based screening programs and heightened awareness efforts over the years. According to the National Family Health Survey Round 5, the screening prevalence for women in Kerala is 3.5%, which is higher than in many other North Indian states [10]. The higher screening proportions in Kerala may be due to better educational levels and greater accessibility to healthcare facilities.

We found that women above 41 years and those who received health information from healthcare workers about CC were more likely to be screened. A study done in Kolkata [22] also showed that advancing age and good knowledge are associated with better uptake of CC screening. This trend may be due to increased awareness of screening importance with age or because older women tend to visit more often for health check-ups and undergo opportunistic testing.

The significant association between APL category and CC screening uptake is also consistent with previous research. A study done on district-level analysis of utilization of CC screening in India also found that women belonging to higher SES groups were more likely to undergo screening for CC [11]. Women in higher SES groups often have more financial resources and social support, which enables them to prioritize and access preventive healthcare services more effectively [23].

The study revealed that 83.4% of participants had heard about CC, a notably higher rate compared to studies from North India [24] and South India [25]. However, nearly half of the study participants were unaware of risk factors for CC, and among those who were aware, only one-fourth identified HPV as a risk factor. A study from Bihar reported that only 15% of women recognized HPV infection as a risk factor [26]. This highlights the need for public health initiatives to spread information about HPV and its link to CC. Despite HPV being a well-established cause of CC [27], the low level of awareness highlights a disconnect between available medical knowledge and public understanding [28,29].

Despite a relatively high awareness of CC, there was a significant gap in knowledge regarding screening tests. To bridge the awareness practice gap and enhance CC screening uptake, it is crucial to implement multifaceted education and awareness programs that are specifically designed to address local needs and involve diverse stakeholders [30]. Additionally, the introduction and scale-up of self-testing options, such as the self-HPV test kit, could be an effective strategy to expand screening in low- and middle-income countries, as these tests are often preferred by women due to their reduced discomfort, embarrassment, and privacy [31].

The study included a relatively large sample size and employed a multistage random sampling technique, enhancing the representativeness of the study population. The limitation of the study is that it relies on self-reported data, which is susceptible to memory and social desirability biases, which could distort knowledge and behavior representation. The findings are context-specific and may have limited generalizability, as awareness and screening behavior are influenced by region-specific factors such as literacy levels and access to health services.

## Conclusions

The present study demonstrates that CC screening uptake among rural women in Ernakulam remains suboptimal despite a relatively high level of awareness. Older age, higher SES, nuclear family type, and receiving information from healthcare workers were independently associated with better screening practices. Strengthening community-based awareness initiatives and enhancing the role of frontline health workers could bridge the knowledge-practice gap. Expanding access through affordable and acceptable strategies such as self-sampling may further support Kerala’s progress toward CC elimination.
